# Chemotherapy-induced peripheral neurotoxicity and complementary and alternative medicines: progress and perspective

**DOI:** 10.3389/fphar.2015.00234

**Published:** 2015-10-23

**Authors:** Xiao L. Cheng, Hong Q. Liu, Qi Wang, Jie G. Huo, Xiao N. Wang, Peng Cao

**Affiliations:** ^1^Affiliated Hospital of Integrated Traditional Chinese and Western Medicine, Nanjing University of Chinese MedicineNanjing, China; ^2^Laboratory of Cellular and Molecular Biology, Jiangsu Province Academy of Traditional Chinese MedicineNanjing, China; ^3^Jiangsu Shenlong Pharmaceutical Co., Ltd.Yancheng, China

**Keywords:** chemotherapy-induced peripheral neurotoxicity, complementary and alternative medicine, herbal medicine, acupuncture, Pathogenesis

## Abstract

Chemotherapy-induced peripheral neurotoxicity (CIPN) is a severe and dose-limiting side effect of antineoplastic drugs. It can cause sensory, motor and autonomic system dysfunction, and ultimately force patients to discontinue chemotherapy. Until now, little is understood about CIPN and no consistent caring standard is available. Since CIPN is a multifactorial disease, the clinical efficacy of single pharmacological drugs is disappointing, prompting patients to seek alternative treatment options. Complementary and alternative medicines (CAMs), especially herbal medicines, are well known for their multifaceted implications and widely used in human health care. Up to date, several phytochemicals, plant extractions, and herbal formulas have been evaluated for their potential therapeutic benefit of preventing the onset and progression of CIPN in experimental models. Clinical acupuncture has also been shown to improve CIPN symptoms. In this review, we will give an outline of our current knowledge regrading the advanced research of CIPN, the role of CAMs in alleviating CIPN and possible lacunae in research that needs to be addressed.

## Introduction

Chemotherapy-induced peripheral neurotoxicity (CIPN) is a severe adverse effect of antineoplastic drugs like taxanes, platinum drugs, vinca alkaloids as well as proteasome inhibitors bortezomib (Miltenburg and Boogerd, 2014). These regimens affect sensory nerves and lead to slow action potential, considerable pain, functional loss, and ultimately chemotherapy withdrawal (Jaggi and Singh, 2012). Generally, CIPN is characterized by pain, tingling, numbness, and impaired sensory function in hands and feet (Miltenburg and Boogerd, 2014). In some cases, motor nerves and autonomic nervous system may also be involved, depending on the antineoplastic agents used (Argyriou et al., 2014). Over the past decades, many valuable strategies such as OPTIMOX (stop and go) have been proposed for CIPN prevention (Hershman et al., 2014). However, dose reduction or cessation can increase cancer-related morbidity and mortality. Hence, an alternative or novel approach is required to treat or prevent CIPN.

Complementary and alternative medicine (CAM), differing from medical mainstream, is historic and widely utilized to treat health conditions throughout the world. Based on recent literatures, several CAM methods exhibiting promising effects on CIPN or a putative influence on mechanisms of CIPN have been identified. Due to its multilevel, multitarget, and coordinated intervention effects, CAM seems to be a promising and viable choice for CIPN prevention. In this review, we will focus on the new insights on the molecular mechanisms of CIPN, and highlight the importance of CAM in alleviating CIPN. Additionally, the strategies for the future research are also proposed in this paper.

## Pathogenesis Of CIPN

**FIGURE 1:**
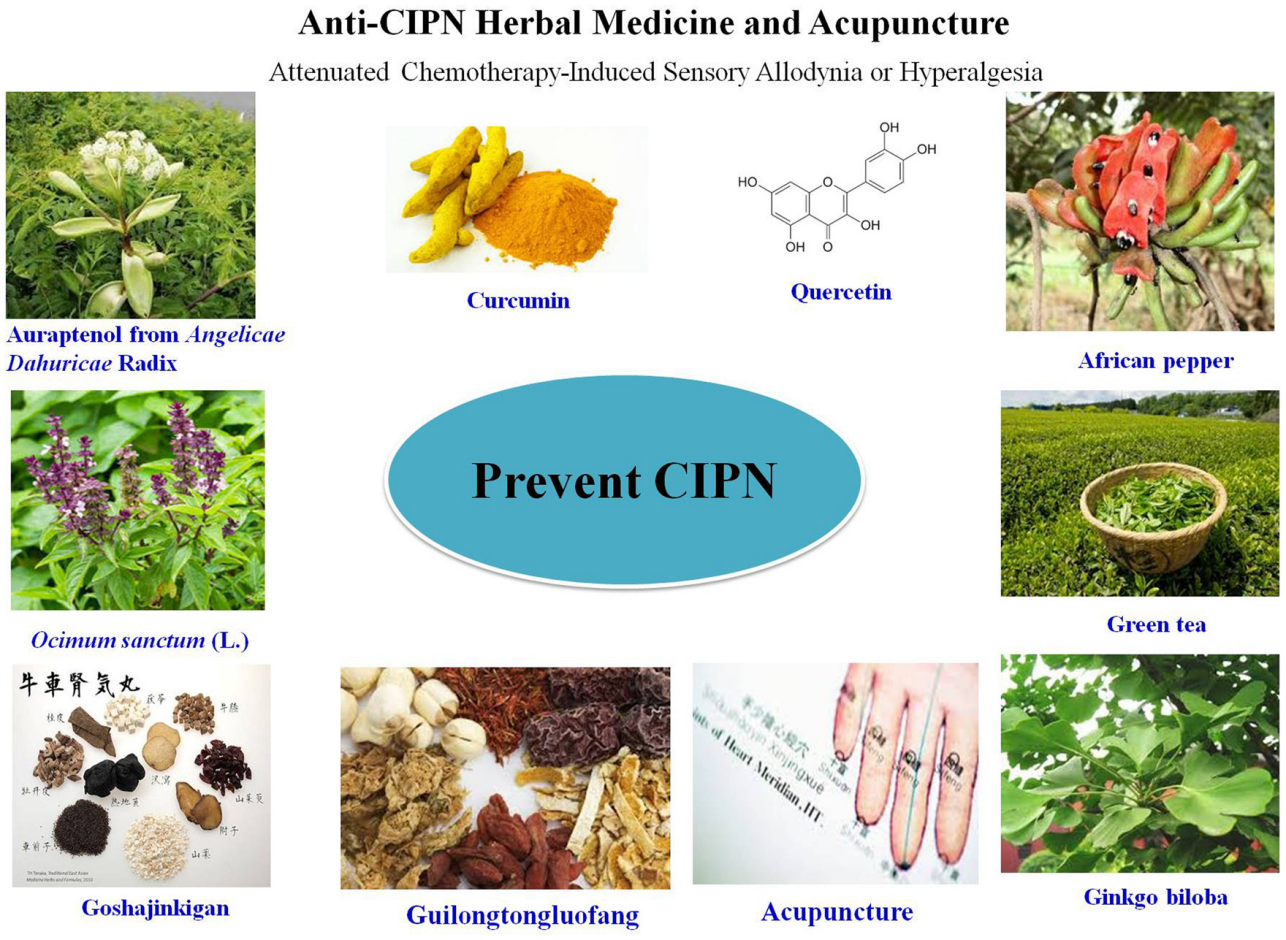
**Summary of the mechanisms of chemotherapy-induced peripheral neurotoxicity (CIPN) and experimental evidence-based applications of complementary and alternative medicines (CAMs) for its prevention or treatment.** All the icons in this figure were obtained from http://image.baidu.com.

Although CIPN have been well explored with the advent of rodent models over the past decade, its exact pathogenesis still remains unclear (Han and Smith, 2013; Carozzi et al., 2015). Recent studies showed that multiple mechanisms including structural changes in peripheral nerves, DNA damage, mitochondria changes, increased oxidative stress, alterations in ion channels, and neuroinflammation activation contributed to the peripheral neurotoxicity development (**Figure 1**, Miltenburg and Boogerd, 2014; Carozzi et al., 2015). In this paper, the main mechanisms involving in CIPN development were reviewed.

### Structural Changes in Peripheral Nerves

Peripheral nervous system is susceptible to the neurotoxins accumulation due to the absence of vascular barrier and lymph drainage. After exposured to chemotherapeutic agents, the longest axons and myelinated fibers are damaged, accompanying reduced sensory nerve conduction velocity and intraepidermal nerve fiber (IENF) loss (Bennett et al., 2011; Boyette-Davis et al., 2011; Zheng et al., 2012). Peripheral nerve degeneration or progressive IENF loss are considered as the key neuropathologies of CIPN. Usually, the structural changes in peripheral nerves may lead to the development of clinical symptoms in the feet and hands, as described as a “stocking and glove” distribution (Han and Smith, 2013). The decrease in IENF density is correlated with the severity of painful neuropathy and hyperexcitability. In CIPN, Aδ (cool specific) and C fibers (warm specific) losses are also observed from nociceptors, resulting in cold-allodynia (Polomano et al., 2001; Flatters and Bennett, 2004). In general, the structural damage extent of peripheral nerves depends on the type of antineoplastic drugs and the dosing regimen, as needs to be further investigated systematically and confirmed in the rodent models.

### Mitochondrial Dysfunction and Oxidative Stress

Accumulating evidences suggest mitochondrion is a key target of CIPN (Poratz et al., 2011; Zheng et al., 2012). After exposure to toxic concentrations of antineoplastic drugs, a reduction in functional mitochondria and a loss of mitochondrial membrane potential and ultra-structural changes were observed in cultured DRG sensory neurons, suggesting subcellular vacuolar degeneration (Melli et al., 2008). *In vivo* studies, histological observations on peripheral nerve of CIPN animals show swollen and vacuolated mitochondria (Melli et al., 2008). The incidence of vacuolated mitochondria in sensory nerve fibers of paclitaxel- or oxaliplatin-treated rats are greatly higher than that in vehicle control group (37.3 and 152%, respectively; Xiao and Bennett, 2012). In patients with CIPN induced by vincristine and bortezomib, the expression of genes controlling the mitochondrial function is significantly changed (Broyl et al., 2010). Anticancer drugs induce mitochondria damage mainly through impairments of ATPase-dependent Na/K pumps and calcium homeostasis alterations. Reducing mitochondrial impairment or suppressing mitochondrial electron transport chain and ATP synthesis was shown to attenuate neurotoxicity symptoms, supporting the important role of mitochondrion in CIPN development (Melli et al., 2008). Accumulation of dysfunctional mitochondria would lead to an increase in oxidative stress, which is also involved in peripheral nerve damage (Sandireddy et al., 2014). In CIPN animals, oxidative stress markers such as oxidative lipid, protein, and DNA damage are dramatically increased in sciatic nerve and lumbar spinal cord (Florea and Büsselberg, 2011; Wang et al., 2011; Di Cesare et al., 2012). Compounds with antioxidant property are demonstrated to relieve the CIPN symptoms (Fidanboylu et al., 2011; Kim et al., 2011). Recently, Nrf2 and NF-κB have been revealed to be co-ordinated for maintenance of redox homeostasis in healthy cells (GaneshYerra et al., 2013). A decline in Nrf2 activity and a persistent increase in NF-κB activity can lead to neuroinflammation and increase oxidative stress, which further result in the development of peripheral neuropathy (GaneshYerra et al., 2013). Hence, agents that can regulate the crosstalk between Nrf2 and NF-κB might be promising to prevent or treat CIPN (Negi et al., 2011).

### Ion Channels

Ion channels including voltage gated Na^+^ and TRP channels have significant roles in CIPN development (Goswami, 2012; Argyriou et al., 2013). Changes in Na^+^ channel induce ectopic activity in primary afferent neurons and result in paraesthesia and fasciculations (Webster et al., 2005). In a previous study, oxaliplatin was found to increase Na^+^ current in DRG neurons. However, in another work oxaliplatin slowed inactivation kinetics of Na^+^ channel, shifted the voltage dependence of gating, and reduced overall Na^+^ current (Sittl et al., 2012). Paclitaxel-induced peripheral neuropathy is also associated with Na^+^ channels (Zhang et al., 2014). Tetrodotoxin, a Na^+^ channel blocker, was able to ameliorate paclitaxel-induced pain (Nieto et al., 2008). Besides Na^+^ channels, transient receptor potential channels such as TRPV1, TRPA1, and TRPM8 play a pivotal role as sensors for cold, mechanical (TRPA1 channels) and heat (TRPV1 channels) stimuli in CIPN models (Goswami, 2012; Hara et al., 2013; Sałat et al., 2013; Quartu et al., 2014). Cisplatin or oxaliplatin can increase expression of TRPA1, TRPM8, and TRPV1 mRNA in DRG neurons. TRPV1 is essential for the generation of thermal hyperalgesia caused by cisplatin (Gauchan et al., 2009a; Anand et al., 2010). Compared to wild-type mice, only mechanical allodynia without heat-evoked pain responses is observed in cisplatin-treated TRPV1-null mice (Ta et al., 2010). Oxaliplatin induces neuropathy partly through regulating TRPA1 and TRPM8 (Gauchan et al., 2009b). Administration of ADM-09, a TRPA1 blocker, is able to effectively abolish oxaliplatin-induced neurotoxicity in mice (Nativi et al., 2013). Besides TRPV1, TRPA1, and TRPM8, TRPV4 may be involved in chemotherapy-evoked peripheral neuropathy. In vincristine- or paclitaxel-treated mice lacking TRPV4, the occurrence of mechanical hyperalgesia was significantly reduced (Alessandri-Haber et al., 2008). Moreover, after spinal intrathecal administration of antisense oligodeoxynucleotides to TRPV4, the reduction of mechanical hyperalgesia was also observed. To date, studies of TRP channels remains limited and should be extended for seeking novel therapeutic strategies to management CIPN.

### Neuroinflammation

Chemotherapy-induced peripheral neurotoxicity development is accompanied by a neuroinflammatory response. Once chemotherapy-induced injury occurs, numbers of inflammatory cells accumulate around damaged nerves, in response to the activation of Schwann cells and resident macrophages, and produce multiple cytokines and chemokines, such as TNF-α, IL-1β, IL-6, IL-8, CCL2, and CXC family. These secreted inflammatory mediators can up-regulate the expression levels of ion channels like Na^+^ and Ca^2+^, or directly activate nociceptors implicated in mechanical and thermal hyperalgesia, and cause peripheral sensitization (Schafers and Sorkin, 2008; Mangiacavalli et al., 2010; Wagner et al., 2011). Studies conducted by Ledeboer and his collaborators demonstrated an increase in pro-inflammatory cytokine gene expressions in paclitaxel-treated lumbar DRG (Ledeboer et al., 2007). Inhibition of inflammatory cytokines has been considered as a useful method for CIPN prevention (Wolf et al., 2006). In an animal model of paclitaxel-induced neuropathy, the mechanical allodynia response could be significantly reversed after IL-10 gene therapy through reducing the production of IL-1β and TNF-α in the DRG (Ledeboer et al., 2007). Toll-like receptors TLR2 and TLR4 in periphery may be involved in mechanical allodynia associated with anticancer drugs. Once stimulated with neurotoxic compounds, TLR2 and TLR4 were activated and then initiated inflammation and caused the elevation of proinflammatory cytokines (Akira and Takeda, 2004). Intrathecally delivered TLR4 receptor antagonists reversed the established mechanical allodynia evoked by paclitaxel (Hutchinson et al., 2008).

### Drug Transporters

A recent study suggested that the neurotoxicity of platinum drugs was correlated with several classes of drug transporters (Ceresa and Cavaletti, 2011). Copper transporters (CTR1) and organic cation transporters (OCT2) have been recognized to be responsible passage for platinum drugs entering into DRG neurons (Ciarimboli et al., 2010; Liu et al., 2013). CTR1 and OCT2 expression was confirmed in the DRG neurons (Cavaletti et al., 2014). OCT2 overexpress can largely improve the uptake of oxaliplatin by 16- to 35-fold (Sprowl et al., 2013). In OCT2-knockout mice, oxaliplatin-induced cold hypersensitivity or mechanical allodynia were totally reversed, suggesting that oxaliplatin-induced peripheral neurotoxicity is dependently mediated by drug transporters, expecially OCT2. So far, knowledge about distribution and activity of platinum drug-related transporters are still very limited, the co-expressions of these different drug transporters and their interplay should be carefully assessed.

## Treatment Options for CIPN and Limitations

So far a variety of pharmacological strategies have been tested to improve the neurological symptoms of CIPN. These promising drugs include PARP inhibitors, Ca/Mg, vitamin E, amifostine, glutathione, glutamine, *N*-acetylcysteine, acetyl-L-carnitine, recombinant human leukemia inhibitory factor, and venlafaxine (Flatters et al., 2006; Cavaletti, 2011; Gobran, 2013; Ta et al., 2013). Although these medications have been proven to be effective in preventing CIPN, their therapeutic potential is limited due to contradictive conclusions and unexpected side effects (Wolf et al., 2008; Ceresa and Cavaletti, 2011). For example, Ca/Mg decreased neuropathy by about 50% compared with a historical control group (Gobran, 2013). However, Ca/Mg can interfere with the response to oxaliplatin-based chemotherapy, and is shown to be ineffective in a large phase III clinical trial (Gamelin et al., 2008; Loprinzi et al., 2014). Glutathione is useful for preventing CIPN in patients undergoing cisplatin-based chemotherapy (Leal et al., 2014). But glutathione may also diminish the antitumour activity of cisplatin through increasing the elimination of cisplatin from kidney (Wolf et al., 2008). To date, no agent has available evidence sufficient to recommend its clinical use for CIPN treatment.

## Complementary and Alternative Medicines for CIPN Treatment

The lack of effectively curative strategies for CIPN promotes the urgent need to seek help from CAM. As a key complement for conventional medical therapy, CAM has been paid attention by the western country because of its less invasive, safe, effective, economical, and convenient therapeuticals benefits. CAM emphasizes on both disease prevention and treatment and has become an important method in treating chronic disease. Most recently, several CAM methods including traditional herbal medicines and acupuncture have been described to be beneficial on CIPN. In present review, clinical and experimental evidence supporting CAMs application for CIPN treatment have be summarized with a special focus on herbal medicines (**Figure 1** and **Table 1**).

**Table 1:** Summary of the proved effects of herbal medicines in chemotherapy-induced peripheral neurotoxicity (CIPN) model and neuropathy symptoms.

Herbal medicines	Dose	Animal model	Mode of action	Reference
*Ginkgo biloba*	100 mg/kg	Cisplatin-induced CIPN in mice	Preventing the reduction in NCV, number of migrating cells, and length of outgrowing axons caused by cisplatin	Lee et al., 2012
	50–150 mg/kg	Vincristine-induced CIPN in rats	Increased the paw withdrawal threshold to mechanical stimuli, reduced withdrawal frequency to cold stimuli	Park et al., 2012
Green tea	300 mg/kg	Oxaliplatin-induced CIPN in rats	Alleviate sensory symptoms such as allodynia, but did not prevent morphometric or electrophysiological alterations induced by oxaliplatin	Wang et al., 2008
*Ocimum sanctum* (L.)	100–200mg/kg	Vincristine-induced CIPN	Attenuated vincristine-induced painful neuropathic state along with decrease in oxidative stress and calcium levels	Kaur et al., 2010
*Matricaria chamomilla*	25 mg/kg	Cisplatin-induced CIPN	Decrease of pain responses in the first and second phase	Namvaran Abbas Abad et al., 2011
*Butea monosperma*	400 mg/kg	Vincristine-induced CIPN	Attenuated vincristine-induced painful behavioural, histopathological changes and alterations of oxidative stress marker	Thiagarajan et al., 2013
Walnut	6%	Cisplatin-induced CIPN in rats	Improved memory and motor abilities in cisplatin treated rats, reduced latency of response to nociception	Shabani et al., 2012
*Xylopia aethiopica*	30–300 mg/kg	Vincristine-induced CIPN	Exhibited anti-hyperalgesic, tactile, and cold anti-allodynic properties	Ameyaw et al., 2014
Curcumin	10 mg/kg	Oxaliplatin and cisplatin neurotoxicity in rats	Reversed the alterations in the plasma neurotensin and sciatic nerve platinum concentrations, and markedly improved sciatic nerve histology in the platinum-treated rats	Al Moundhri et al., 2013
Auraptenol	0.05–0.8 mg/kg	Vincristine-induced CIPN in mice	Dose-dependently reverted the mechanical hyperalgesia	Wang et al., 2013
Quercetin	50 mg/kg	Oxaliplatin-induced CIPN in mice	Prevented oxaliplatin induced painful peripheral neuropathy, prevented lipid peroxidation and tyrosine nitrosylation	Azevedo et al., 2013
Goshajinkigan	0.3–1 g/kg	Oxaliplatin-induced CIPN in rat	Prevent oxaliplatin-induced cold hyperalgesia but not mechanical allodynia and axonal degeneration of the rat sciatic nerve	Andoh et al., 2014
	1 g/kg	Paclitaxel-induced CIPN in mice	Prevent paclitaxel-induced allodynia without affecting the anticancer action	Kono et al., 2011
Guilongtongluofang	200 mL/day	A randomized, double-blind, placebo-controlled trial	Reduce the incidence of neurotoxicity without reducing the efficacy of chemotherapy	Liu et al., 2013

### Herbal Medicines

#### Curcumin

Curcumin is the major active ingredient of turmeric and ginger, with strong antioxidant and anti-inflammatory activities. Curcumin has been shown to be a neuroprotective agent against neurological disorders, including diabetic neuropathy and alcoholic neuropathy (Ataie et al., 2010; Attia et al., 2012; Kandhare et al., 2012). In CIPN rat model, curcumin reduced plasma neurotensin and platinum uptake in sciatic nerve, and profoundly improved histopathological damages induced by oxaliplatin and cisplatin (Al Moundhri et al., 2013). In PC12 cells, curcumin could largely reversed the cisplatin-induced reduced neurite outgrowth of cells, without compromising anticancer activity (Mendonça et al., 2013). Curcumin ameliorated altered non-enzymatic and enzymatic antioxidants and complex enzymes of mitochondria, thus holding promise as agent that can potentially reduce platinum -induced peripheral neurotoxicity (Waseem and Parvez, 2015).

#### Quercetin

Quercetin is a flavonoid widely distributed in many plants and fruits including *Bupleurum chinense* DC., *Morus alba* L., *Crataegus pinnatifida* Bunge, red grapes, and citrus fruit, and has been reported to have powerful antioxidant, antinociceptive as well as anti-inflammatory properties. With several animal models, this compound showed remarkable antinociceptive and neuroprotective effects in alcohol and diabetic induced neuropathies (Wang et al., 2011; Raygude et al., 2012) threshold, prevented the shrinkage of neurons and inhibited light edema formation (Azevedo et al., 2013). Additionally, marker of neuroplasticity c-Fos was lower in quercetin pretreatment groups (25, 50, and 100 mg/kg) than that of oxaliplatin-treated rats. The action mechanism of quercetin is associated with its attenuation of mitochondrial dysfunction induced by oxaliplatin (Waseem and Parvez, 2015).

#### *Ginkgo biloba* Extract

*Ginkgo biloba* extract (GBE), the leaf extract of *Ginkgo biloba* L., is a popular herbal product used for a variety of ischemic and neurological disorders. Growing studies have reported the antioxidant, anticancer, angiectatic, and neuroprotective potentials of GBE. The evidence for the protective role of GBE in ameliorating CIPN is also available in several *in vivo* studies (Oztürk et al., 2004; Park et al., 2012). In mice with peripheral neuropathy induced by cisplatin, oral administration of GBE (100 mg/kg/d) for 4.5 weeks was demonstrated to promote axonal growth from DRG, and prevent the reduction in sensory nerve conduction velocity. Furthermore, reductions of length of outgrowing axons, and somatic and nuclear sizes of neurons were also reversed (Oztürk et al., 2004). In a rat model of vincristine-induced peripheral neuropathy, GBE significantly increased the paw-withdrawal threshold to mechanical stimulation and reduced withdrawal latency to cold stimuli (Park et al., 2012). The antihyperalgesic effect of GBE may be associated with its antioxidative actions, suppression of NF-κB, NO, and TNF-α production, inhibition of myelinated axons degradation and improvement of axonal transport.

#### Green Tea

Green tea is a popular beverage with attractive flavor, aroma, and health effect. The major bioactive compounds presented in green tea are catechins. Numerous published studies have reported that catechins possess potent antioxidant and anti-inflammatory activities, and have been shown to prevent cancer and improve chemotherapy-induced side effects (Zaveri, 2006). The beneficial effect of green tea in ameliorating experimental CIPN was assessed in oxaliplatin-treated rats (Lee et al., 2012). Coadministration of green tea at 300 mg/kg for 6 weeks effectively alleviated mechanical allodynia and thermal hyperalgesia induced by oxaliplatin. However, the exact mechanisms responsible for antiallodynic and antihyperalgesic activity of green tea are not clear.

#### Goshajinkigan

Goshajinkigan (GJG) is a widely used Kampo medicine containing 10 different herbs (*Rehmannia viride radix, Achyranthis bidentatae radix, Corni fructus, Dioscorea opposita rhizoma, Plantaginis semen, Alismatis rhizoma, Moutan cortex, Cinnamomi cortex, Aconiti lateralis praeparata tuber, and Poria alba*). Prescription of GJG to diabetic patients can improve neuropathy symptoms such as numbness, cold sensation, and limb pain (Uno et al., 2005). In recent years, the effect of GJG on CIPN has been extensively explored. In CIPN rats, GJG treatment was able to reduce cold hyperalgesia and mechanical allodynia, and no regeneration was found in histological examination (Ushio et al., 2012; Bahar et al., 2013; Andoh et al., 2014). More importantly, GJG showed little effect on the antitumour activity of anticancer drugs. The neuroprotection of GJG has also been supported by clinical studies. In a retrospective analysis included 45 patients with colorectal cancer, 22 received GJG during their FOLFOX regimen, while 23 did not get this additional therapy. After 10 courses of chemotherapy, the prevalence of grade 3 peripheral neuropathy in the GJG group was 0%, while 12% in the control group. After 20 courses, the incidence in the GJG group increased to 33%, significantly lower than that in patients without GJG administration (75%; Kono et al., 2011; Nishioka et al., 2011). Results from large clinical trials enrolling patients with colorectal, breast, and gynecological cancers further support the protective effect of GJG (Yamamoto et al., 2009). Although GJG is effective for treating CIPN, its underlying molecular and cellular mechanisms remain poorly understood. Several laboratory studies indicated that GJG improved peripheral nociception and circulation through promoting NO production, increasing hippocampal c-Fos and nerve growth factor expression, and suppressing functional alteration of TRP channels such as TRPA1 and TRPM8 (Yamamoto et al., 2009; Mizuno et al., 2014).

#### Other Herbal Medicines

In addtion to the phytochemicals and herbs mentioned above, several other compunds or herbal mixtures also presented positive effect on CIPN treatment. For example, auraptenol, a coumarin component isolated from Angelicae Dahuricae Radix, was reported to protect mice from vincristine-induced neuropathic pain (Wang et al., 2013). After auraptenol treatment (0.8 mg/kg), mechanical hyperalgesia was totally suppressed. African pepper may inhibit p38 and/or ERK1 and ERK2 pathways, and prevent pain stimuli propagation in the degenerated C-, Aδ-, and Aβ-fibers, thereby reversing mechanical hyperalgesia and cold allodynia (Ameyaw et al., 2014). In vincristine-induced neurotoxicity of rat model, *Ocimum sanctum* (L.) lowered the level of oxidative stress and calcium, thus helping to prevent CIPN symptoms (Kaur et al., 2010). Guilong tongluo formula (GLTLF) has also been shown to reduce CIPN symptoms. After four cycles of treatment, the percentage of neurotoxicity in GLTLF-treated group was 51.7% compared with 70.0% for placebo-treated group (Liu et al., 2013). In addition, the onset of sensory neurotoxicity was much later in patients who received GLTLF.

### Acupuncture

Cancer patients often seek CAM help for treatment-related side effects. Acupuncture, stimulating the special body points by the thin needles, is one of the most frequently used remedies, and effective for various adverse reactions resulting from chemotherapy or radiation therapy (Dean-Clower et al., 2010). Recently, acupuncture has been tested for CIPN in experimental models and clinical trials (Schroeder et al., 2012). The results demonstrated that intervention with acupuncture can increase limb blood flow, promote nerve repair, inhibit peripheral nerves degradation and induce a normalization of histological morphology (Litscher et al., 2002; Xu et al., 2010). The benifical role of acupuncture on CIPN may be mediated by the enhancement of spinal/central GABA-ergic, serotoninergic, and adrenergic neurotransmission, as well as the parallel decrease in sensory neurons hypersensitization (Park et al., 2010; Silva et al., 2011). With cDNA microarray analysis, it was found that the ation of mechanism of acupuncture involved signal translation, gene expression, and nociceptive pathways (Ko et al., 2002). Acupuncture seems promising because of its safety and low cost. According to American College of Chest Physicians evidence-based clinical practice guidelines for lung cancer, complementary acupuncture is recommended when pain is poorly controlled or neuropathy is clinically significant (Cassileth et al., 2007). However, for extensive application in CIPN, the efficacy of acupuncture still needs to be confirmed by more rigorous randomized controlled clinical studies.

## Conclusion and Future Prospects

As a prominent dose-limiting side effect in chemotherapy, CIPN is attached great importance. Due to multiple mechanisms of neuronal demage, a combination of components focusing on multiple targets of CIPN might be promising. Recently, CAM therapies including herbal medicines and acupuncture have been intensively studied for CIPN prevention and show promising results. However, the scientific evidence supporting their efficacy is strikingly limited. Furthermore, the dosages of some herbal medicines used in rodent models seem quite high, and some herbs still cause hepatic or renal toxicity at high dosages. Therefore, it is necessary and important to confirm whether the dosages could be interpretated to clinical settings and determine the toxicity dosage of herbal preparation. Acupuncture in CIPN management has been pomising, yet, the studies quality conducted is low. The postition, depth, and angle of the needle insertion are not cosistent, which need to be improved in interpreting the findings. In summary, when considering CAMs use in the treatment of CIPN, the therapeutic potential of alternative therapies still needs to be rigorously investigated with large scale randomized controlled trials. Additionally, the interactions of CAMs with chemotherapy, potential toxicities associated herb medicines, as well as molecular mechanisms and bioactive compounds responsible for the neuroprotective effects should also be further investigated.

## Author Contributions

All authors fulfil the authorship requirements and have approved the final version of the manuscript. PC, XW, and JH developed the paper design and revised the manuscript. QW contributed to revise the manuscript. XC and HL wrote the first draft of the manuscript to which all authors made significant subsequent contributions.

## Conflict of Interest Statement

The authors declare that the research was conducted in the absence of any commercial or financial relationships that could be construed as a potential conflict of interest.
